# PncsHub: a platform for annotating and analyzing non-classically secreted proteins in Gram-positive bacteria

**DOI:** 10.1093/nar/gkab814

**Published:** 2021-09-22

**Authors:** Wei Dai, Jiahui Li, Qi Li, Jiasheng Cai, Jianzhong Su, Christopher Stubenrauch, Jiawei Wang

**Affiliations:** School of Computer Science and Information Security, Guilin University of Electronic Technology, Guilin 541004, China; Infection and Immunity Program, Biomedicine Discovery Institute and Department of Microbiology, Monash University, VIC 3800, Australia; Wenzhou Institute, University of Chinese Academy of Sciences, Wenzhou 325011, China; School of Computer Science and Information Security, Guilin University of Electronic Technology, Guilin 541004, China; School of Computer Science and Information Security, Guilin University of Electronic Technology, Guilin 541004, China; School of Computer Science and Information Security, Guilin University of Electronic Technology, Guilin 541004, China; Wenzhou Institute, University of Chinese Academy of Sciences, Wenzhou 325011, China; School of Ophthalmology & Optometry and Eye Hospital, Wenzhou Medical University, Wenzhou 325027, China; Infection and Immunity Program, Biomedicine Discovery Institute and Department of Microbiology, Monash University, VIC 3800, Australia; Centre to Impact AMR, Monash University, VIC 3800, Australia; Infection and Immunity Program, Biomedicine Discovery Institute and Department of Microbiology, Monash University, VIC 3800, Australia; Centre to Impact AMR, Monash University, VIC 3800, Australia

## Abstract

From industry to food to health, bacteria play an important role in all facets of life. Some of the most important bacteria have been purposely engineered to produce commercial quantities of antibiotics and therapeutics, and non-classical secretion systems are at the forefront of these technologies. Unlike the classical Sec or Tat pathways, non-classically secreted proteins share few common characteristics and use much more diverse secretion pathways for protein transport. Systematically categorizing and investigating the non-classically secreted proteins will enable a deeper understanding of their associated secretion mechanisms and provide a landscape of the Gram-positive secretion pathway distribution. We therefore developed PncsHub (https://pncshub.erc.monash.edu/), the first universal platform for comprehensively annotating and analyzing Gram-positive bacterial non-classically secreted proteins. PncsHub catalogs 4,914 non-classically secreted proteins, which are delicately categorized into 8 subtypes (including the ‘unknown’ subtype) and annotated with data compiled from up to 26 resources and visualisation tools. It incorporates state-of-the-art predictors to identify new and homologous non-classically secreted proteins and includes three analytical modules to visualise the relationships between known and putative non-classically secreted proteins. As such, PncsHub aims to provide integrated services for investigating, predicting and identifying non-classically secreted proteins to promote hypothesis-driven laboratory-based experiments.

## INTRODUCTION

Bacteria are commonly differentiated by the Gram stain reaction according to the structural properties of their cell envelope (1). A negative reaction means the bacterium has a relatively small amount of peptidoglycan, and roughly corresponds to those bacteria surrounded by two membranes. A positive reaction instead means the bacterium has a much thicker peptidoglycan layer and generally means the bacterium is bounded by a single membrane. Gram-positive bacteria are among some of the most industrially and clinically important bacteria, from lactic acid bacteria that are essential for the production of fermented dairy products (e.g. cheese, sour cream, yoghurt) to clinically important multi-drug resistant strains of *Enterococcus faecium* and *Staphylococcus aureus* (2,3). The success of these bacteria is in part due to their large repertoire of non-classical secretory pathways that range in function from cell-to-cell communication, nutrient acquisition, motility, and even pathogenesis.

By far the most important protein secretion apparatus is the general secretion (Sec) machinery, not just because the majority of secreted proteins use this pathway directly, but because other secretion apparatuses are typically inserted into the inner membrane in a Sec-dependent manner (4). Proteins targeted to the Sec machinery must be translocated in an unfolded state due to the narrow confines of the Sec translocation pore (4). For proteins that must first pre-fold in the bacterial cytoplasm, due to the requirement for cytoplasmic cofactors for example, the twin-arginine translocation (Tat) machinery is alternatively used (5). Collectively, the Sec and Tat machines are considered classical secretion systems. They are conserved throughout bacteria and archaea, as well as all eukaryotes (Sec only) or plant thylakoids (Tat only) and their substrates are easily recognised by their highly conserved N-terminal signal sequences (4,5) and readily predicted using any number of webservers, including TMHMM, Phobius and SignalP (6–8).

**Figure 1.**
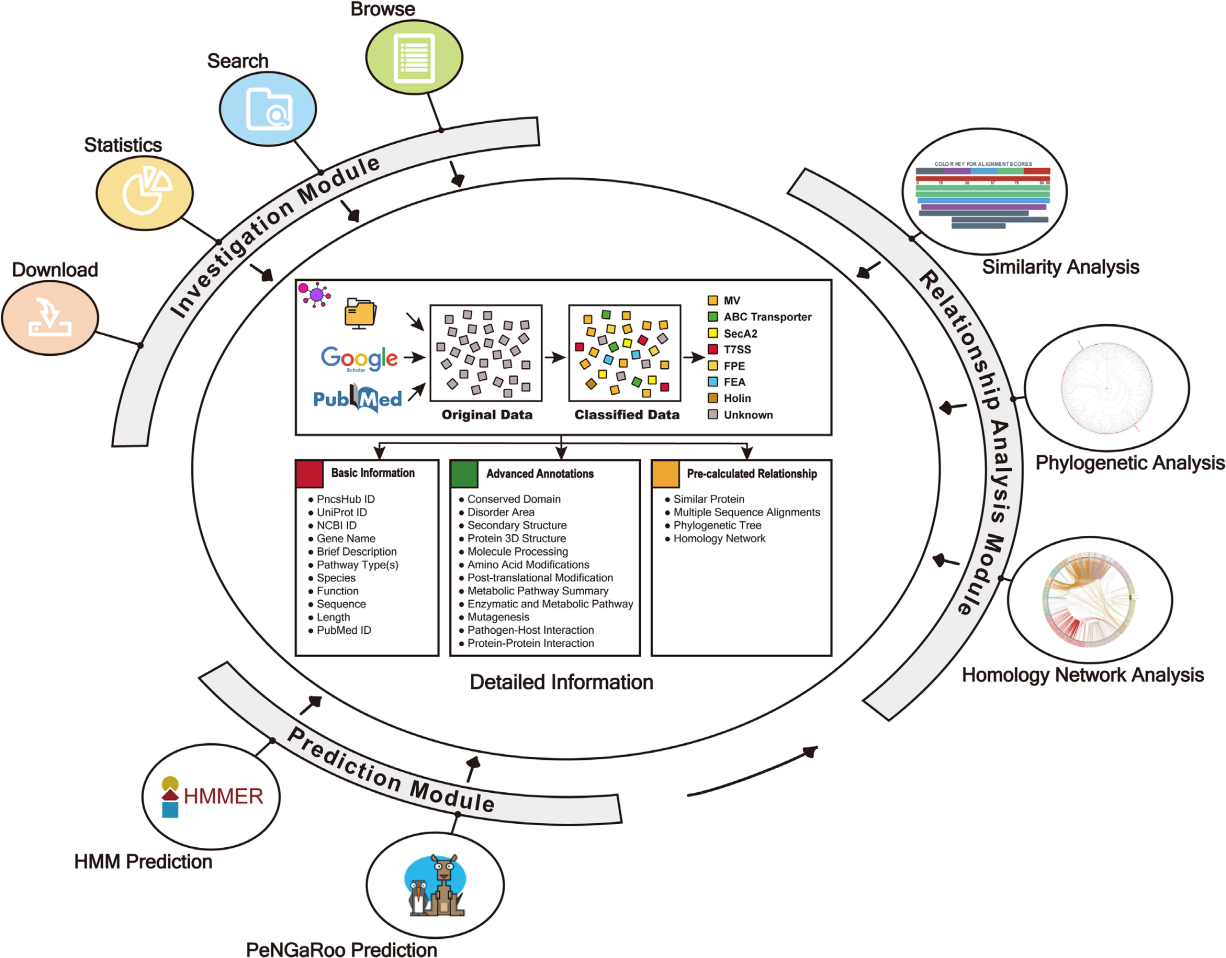
The interconnected modules of PncsHub. PncsHub houses 3 modules for protein investigation, prediction, and analysis. All modules can redirect users to the ‘Detailed Information’ pages comprising complete annotations of experimentally validated non-classically secreted proteins. Additionally, the Prediction and Relationship Analysis modules are connected to allow users to analyze their prediction results to infer likely secretion pathways and generate visually appealing images.

While all bacteria encode the classical translocation machineries, Gram-positive bacteria have evolved at least seven non-classical secretion pathways: ATP-Binding Cassette (ABC) transporters, the Fimbrilin-Protein Exporter (FPE), Flagella Export Apparatus (FEA), Holins, Membrane Vesicles (MVs), SecA2, and the Type VII Secretion System (T7SS) (9–15) (for a description of each system, please see the dedicated section within PncsHub: https://pncshub.erc.monash.edu/help.jsp#secretionsystems). Additionally, there are several lines of evidence to suggest that there are many ‘other’ secretion pathways yet to be discovered (16). To date, there are (or have been) five webservers that predict non-classically secreted proteins from Gram-positive bacteria: Secretome P (17,18), SecretP (19), NClassG+ (20), NonClasGP-Pred (21) and PeNGaRoo (22). Of these, both SecretP and NClassG+ appear to have been decommissioned based on their webservers no longer responding. While SecretomeP has the claim to fame of being the very first predictor for non-classical proteins, it has purportedly fallen out of use considering both its age and unacceptably high false positive rate (22–24). Of the two most recent webservers, PeNGaRoo and NonClasGP-Pred, both have opted for a binary output (yes/no) and are therefore not capable of determining which non-classical secretion system the protein is likely secreted by. Here, we describe the Gram-positive non-classically secreted (Pncs) protein Hub: PncsHub (we pronounce it ‘Pinks Hub’) (Figure 1), a companion database to BastionHub (25), which catalogues Gram-negative non-classically secreted proteins instead. PncsHub combines high quality prediction algorithms, catalogues experimentally verified proteins, and provides a series of data analysis and visualisation tools that can all be used to facilitate both discovery and annotation, and ultimately allow users to determine which secretion pathway a putative substrate likely uses.

## MATERIALS AND METHODS

### Data curation and annotation

**Figure 2.**
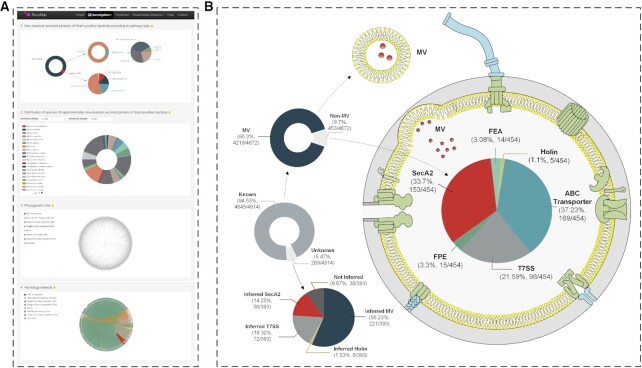
Breakdown of non-classically secreted proteins by sub-type. (**A**) PncsHub Statistics page from within the Investigation Module. (**B**) Overview of non-classically secreted proteins with a known or unknown pathway (left, middle). The known pathways are further divided into membrane vesicles (MVs) and other sub-types (left, upper), of which there are 27 proteins that can be secreted by both MV and one other pathway. The unknown pathway is further annotated with inferred pathways (left, lower). Finally, the other known sub-types are separated into the remaining six sub-types (right): ABC Transporter, flagella export apparatus (FEA), fimbrillin-protein exporter (FPE), Holin, SecA2 (the alternate Sec pathway), and type VII secretion system (T7SS) pathways, and in addition to the 27 overlapping proteins that are also secreted by MV, there is one protein that can be secreted by both T7SS and SecA2.

To date, there are no repositories that document Gram-positive non-classically secreted proteins, so we systematically reviewed existing literature and have accumulated 4914 experimentally verified, non-classically secreted proteins from literature (Figure 2, Supplementary Data S1). Of these, 269 proteins were shown to be secreted but the secretion mechanism was yet to be determined. These proteins were typically identified by immunoblotting and/or proteomics-based investigations of the culture filtrate, without regard for any specific secretion mechanism per se (see, e.g. 26–28). Although classified as ‘Unknown’, in some cases the secretion mechanism is all but certain: consider, for example, the six large Clostridial toxins or bacteriocins encoded adjacent to a Holin gene (29), three have been shown to be secreted through that Holin (11,29–30), whereas the other three secreted proteins have an unknown secretion mechanism. Also, consider the 66 Mycobacterial proteins that are both annotated with an ‘unknown’ secretion mechanism and have conserved domains typified by the Esx, Esp, PE or PPE family proteins. These proteins are generally considered to be secreted by a T7SS, even though this has never been demonstrated experimentally (15). In both cases, although we have annotated them as having an ‘unknown’ secretion mechanism, we have annotated them with an inferred subtype: ‘Possible Holin’ or ‘Possible T7SS’, respectively (Supplementary Data S1). Additionally, we were able to annotate a further 3 ‘Possible Holin’ proteins (PNCS00376, PNCS01173 and PNCS01199), which are exported proteins that are encoded within lysogenic phage loci that are generally accepted to be exported through the Holin encoded nearby (Supplementary Data S1) (26,31).

The vast majority of proteins were identified in MVs (4219 proteins in total) and this obvious bias toward proteins secreted by MVs is easily explained when considering both the identification mechanism and the MVs themselves. Overall, MVs are naturally better capable of secreting a large portion of proteins because they sample a segment of the bacterial cell itself, including lipids, DNA, RNA and proteins (32). Additionally, MVs can be purified from bacterial cells and analysed by proteomics-based methods relatively easily, in fact our database has compiled data from MVs isolated from 29 different Gram-positive bacteria, including bacteria not normally considered genetically tractable. In contrast, secretion through any other pathway typically includes deletion of the pathway of interest, separation of culture filtrate (or cell wall or membranes) from the rest of the cell, and a series of controls to demonstrate secretion wasn’t due to cell lysis.

With the 4914 experimentally verified proteins, we comprehensively annotated the features of each protein. In most cases, UniProt (33) and/or NCBI (34) were used to annotate basic information: gene name, brief description (e.g. protein name), species, function, sequence, and sequence length, as well as UniProt ID (from UniProt only), and the NCBI and PubMed IDs (from NCBI only). Additionally, we manually annotated some proteins with data from the listed references themselves. UniProt was also used to annotate molecule processing details (e.g. location of signal peptides), amino acid modifications (e.g. details about disulphide linkages, unnatural amino acid modifications, etc), post-translational modifications (i.e. a summary of amino acid and molecule processing information), mutagenesis information (i.e. amino acid sites altered experimentally and their corresponding phenotypes), and the metabolic pathway summary. Additionally, further details about enzymatic and metabolic pathways were obtained from BioCyc (35), BRENDA (36), UniPathway (37), Reactome (38) and SABIO-RK (39). The Pfam database (40) was used to annotate Conserved Domain information. ECharts (https://echarts.apache.org/) was used to visualise the natively disordered regions within each substrate, as predicted by the IUPred2A webserver (41,42). The PSIPRED 4.0 server (43) was used to predict and visualise the secondary structure of substrates with less than 1500 residues. Experimentally determined tertiary structure information was obtained from the Protein Data Bank (PDB) (44) and can be visualised using the integrated LiteMol interface (45) if the user clicks the ‘Structure Review’ link. The PHI-base database (46) was used to collect pathogen–host interaction data and protein–protein interactions were obtained from STRING (47), DIP (48), IntAct (49) and/or MINT (50) databases.

Pre-calculated relationship analyses were also included within the detailed information, comprising: Similarity Analysis, Multiple Sequence Alignment, Phylogenetic Analysis and Homology Network Analysis. In each case, the query protein was compared to the other experimentally validated non-classically secreted substrates proteins within PncsHub. For both the Similarity Analysis and Multiple Sequence Alignments, blast 2.8.1+ (51) was used to identify homologous proteins from amongst the experimentally validated non-classically secreted substrate proteins. More specifically, BlasterJS (52) was used to visualize blast alignment results for the Similarity Analysis, whereas the Multiple Sequence Alignment was generated using the ClustalW method (53), which was invoked and visualized using the R Library msa (54). The two remaining visualisation tools incorporated all experimentally validated non-classically secreted substrate proteins. For the Phylogenetic Analysis, the open-source tool phylogram_d3 (https://github.com/ConstantinoSchillebeeckx/phylogram_d3) was used to visualize the phylogenetic tree (without branch length information). The tree was inferred using FastTree (version 2.1.10) (55) from a multiple sequence alignment generated using MAFFT (v7.310) (56). For the Homology Network Analysis, ECharts was used to visualize the homology networks generated using all-against-all BLAST (version blast-2.2.26) (57). Furthermore, if the user hovers over the leaf (in the Phylogenetic tree) or node (in the Homology Network Analysis), the basic information for that protein is displayed (as implemented above). If the user clicks the linked nodes within the Homology Network Analysis, a pair-wise sequence alignment between the linked nodes is displayed, which was generated using the EMBOSS Stretcher web service (58).

### Website architecture and module implementation

PncsHub is based largely on what was previously implemented for AcrHub and BastionHub (25,59) in terms of website design and implementation, unless otherwise indicated. It was implemented as three separate modules: an Investigation Module, a Prediction Module, and a Relationship Analysis Module (Figure 1). In all three modules, users can select from three datasets they want to use for investigation, prediction, and analysis purposes: (i) all data, (ii) all data excluding MV and (iii) MV only data. The annotations incorporated within the Investigation Module are described above. The Prediction Module incorporates three predictors: a lightweight HMM based prediction model developed using HMMER (60), the original PeNGaRoo prediction model (22) (annotated as original PeNGaRoo) and an updated PeNGaRoo prediction model implemented using our current list of experimentally verified non-classically secreted proteins (annotated as retrained PeNGaRoo). In the input page for the Prediction Module, users can select more than one prediction model to be displayed as the final output. In this case, the output page will show each result in tandem on the same page, so users can compare results for each model. Additionally, because the PeNGaRoo predictor was developed using a two-layer LightGBM ensemble model that integrates seven single-feature based models into an overall prediction framework (22), we included each single model prediction score into the retrained PeNGaRoo output. The Relationship Analysis Module (Similarity Analysis, Phylogenetic Analysis, and Homology Network Analysis) was implemented as described above, except that the query sequence is now user-defined within that specific module.

## RESULTS

The overall architecture of PncsHub can be split into three interconnected modules: an Investigation Module, a Prediction Module and a Relationship Analysis Module (Figure 1).

### Investigation module

The Investigation Module comprises a list of fully annotated and experimentally validated non-classically secreted substrates. Users can navigate through the full list using the ‘Browse’ tab, apply filters to the list using the ‘Search’ tab, get an overview of the data from the ‘Statistics’ tab, or obtain a copy of the data using the ‘Download’ tab. The full list of experimentally validated proteins is initially displayed with each protein’s basic information (gene name, description, non-classical pathway, host species), with four clickable IDs: PncsHub ID (which navigates to the ‘Detailed Information’ page), UniProt ID (which navigates to its entry in UniProt (33)), NCBI ID (which navigates to its entry in NCBI (34)), and PubMed ID (which navigates to the relevant publication associated with the entry).

**Figure 3.**
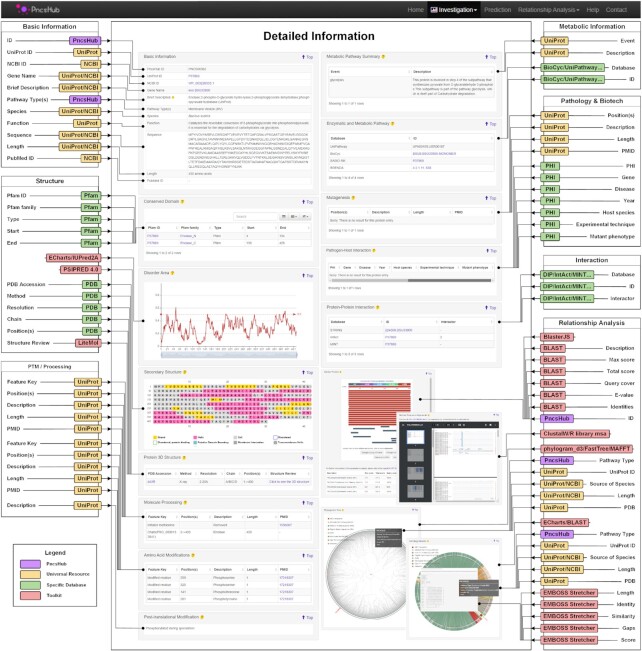
Detailed Information page for enolase (PNCS00362). Each experimentally validated non-classically secreted substrate has been annotated with information from up to 26 webservers, databases and toolkits used to annotate or visualize the data (see the ‘Data curation and annotation’ section for more details about each tool). In some cases, data was extracted by manually inspecting the literature (annotated as PncsHub). Here, we are showcasing the detailed information page for enolase from *B. subtilis* (PNCS00362), which was successfully annotated in all but two categories.

While we have included the major repositories (UniProt, NCBI and PubMed), the ‘Detailed Information’ hosted by PncsHub compiles data from up to 14 more resources and databases, including Pfam, BioCyc, and STRING (see ‘Data Curation and Annotation’ for more details). Using enolase (encoded by *Bacillus subtilis*) as an example (PNCS00362), Figure 3 showcases the ‘Detailed Information’ page, with each of its annotations highlighted. Enolase is usually found in the cytoplasm of bacterial cells and is responsible for converting 2-phosphoglycerate to phosphoenolpyruvate in the penultimate step of the glycolysis pathway. The obvious benefit of our ‘Detailed Information’ pages is that they compile data from a large number of resources in one place and also include pre-calculated relationship analysis data that details each protein's homology information. Indeed, our ‘Detailed Information’ page for enolase (PNCS00362) includes 18 enolase homologues that are secreted from Gram-positive bacteria: 3 with an unknown secretion mechanism (PNCS00297, CPNS00298 and PNCS00299), 14 secreted within MVs from 10 different bacterial genera (PNCS00208, PNCS00648, PNCS01233, PNCS01665, PNCS01828, PNCS02188, PNCS02345, PNCS02434, PNCS03218, PNCS03365, PNCS04117, PNCS04183, PNCS04280 and PNCS04527), and 1 secreted using the SecA2 pathway (PNCS00170) (Supplementary Data S1).

In addition to enolase, we noticed that different bacteria have a preferred secretion mechanism depending on the type of substrate. For example, superoxide dismutase homologues may be secreted via the SecA2 pathway (61,62), the T7SS pathway (63) or through MVs (64,65). Additionally, we found that there were 28 proteins (including superoxide dismutase) that could be secreted through more than one pathway in the same bacterium (Figure 2, Supplementary Data S1). *Bacillus subtilis* may assemble the flagellar components FlgG (PNCS01983), Hag (PNCS00370), and FlgK (PNCS00368) through its FEA apparatus (66–68), or jettison these three components through its MVs (69,70). In *Mycobacterium tuberculosis*, it may secrete any of eight T7SS-dependent effectors through its MVs instead, including the best-studied T7SS effectors: EsxA and EsxB (15,71,72).

### Prediction module

The Prediction Module incorporates three prediction models: a lightweight HMM based predictor and two versions of the PeNGaRoo predictor (22). In each case, PncsHub first determines whether the query sequences are amongst its ‘filter list’ of experimentally validated protein substrates. If they are not experimentally validated (or the user selects the ‘For benchmarking test’ option), the query sequences are fed through to the HMM and/or PeNGaRoo prediction tool. In each case, the predictor returns a binary output (‘yes’/‘no’) as to whether the protein is predicted to be non-classically secreted.

The HMM based prediction is rapid and highly efficient for homologous proteins, but its main drawback is that it is not very sensitive for non-homologous proteins and therefore cannot be used to predict novel substrates. Instead, PeNGaRoo can be used to identify novel substrates. This method makes use of an ensemble learning strategy that extracts different aspects of information from the training dataset (see (22) for more information about this predictor), and is therefore more capable of identifying novel substrates that otherwise appear unrelated to the experimentally validated substrates through sequence identity alone (22). This prediction framework has otherwise been shown to identify highly evolved proteins in bacteria and bacteriophages (73–75). Although the PeNGaRoo predictor is invariably slower than the HMM predictor, it is by far a more powerful technique (see the ‘PncsHub Modules in Action’ section below).

### Relationship analysis module

The Relationship Analysis Module can be used to visualize the similarities (or differences) between query protein(s) and the list of experimentally verified non-classically secreted substrates. We have incorporated three data visualization tools: Similarity Analysis, Phylogenetic Analysis and Homology Network Analysis. These tools can be used as standalone from the relationship analysis tab, but they are much more powerful when used in conjunction with the Prediction Module. This is because the Prediction Module only describe whether the protein is likely to be a non-classically secreted protein, but not which pathway it likely uses. Instead, if users transfer the positive samples to one of the relationship analysis tools, they can potentially infer the likely secretory pathways, as well as potential functions of the protein based on this information (see the ‘PncsHub Modules in Action’ section below).

### PncsHub modules in action

To test the veracity of our methodology, we identified three proteins that are non-classically secreted: EsxB, LF, and SrpC. EsxB is secreted through the ESAT-6 system 1 (ESX-1) T7SS of *M. tuberculosis* and has many homologues across Mycobacteria and other T7SS-containing bacteria (76). LF, one of three anthrax toxin components secreted by *B. anthracis*, is known as lethal factor; it is thought to be secreted through the classical Sec machinery (77) although to our knowledge this hasn’t been specifically demonstrated, but it has otherwise been found to reside within MVs with the two other anthrax toxin components (78). SrpC is one of three serine rich proteins that, due to its extensive post-translational glycosylation, is secreted through the accessory SecA2 system by *Streptococcus salivarius* (79). While we have annotated these proteins in our database (EsxB is PNCS00367, LF is PNCS00598, and SrpC is PNCS04914), none of them were included in the training datasets of the HMM and PeNGaRoo predictors. Additionally, PncsHub stores a built-in and up-to-date ‘filter list’ of experimentally validated non-classically secreted proteins to filter out the query proteins prior to executing its computational prediction (22), and as such, both LF and SrpC were excluded from this list so they would not be filtered out as experimentally verified proteins. As a negative control, we also identified a cytoplasmic protein that is not secreted: KdgA (UniProt ID: P50846) from *B. subtilis* (80).

**Figure 4.**
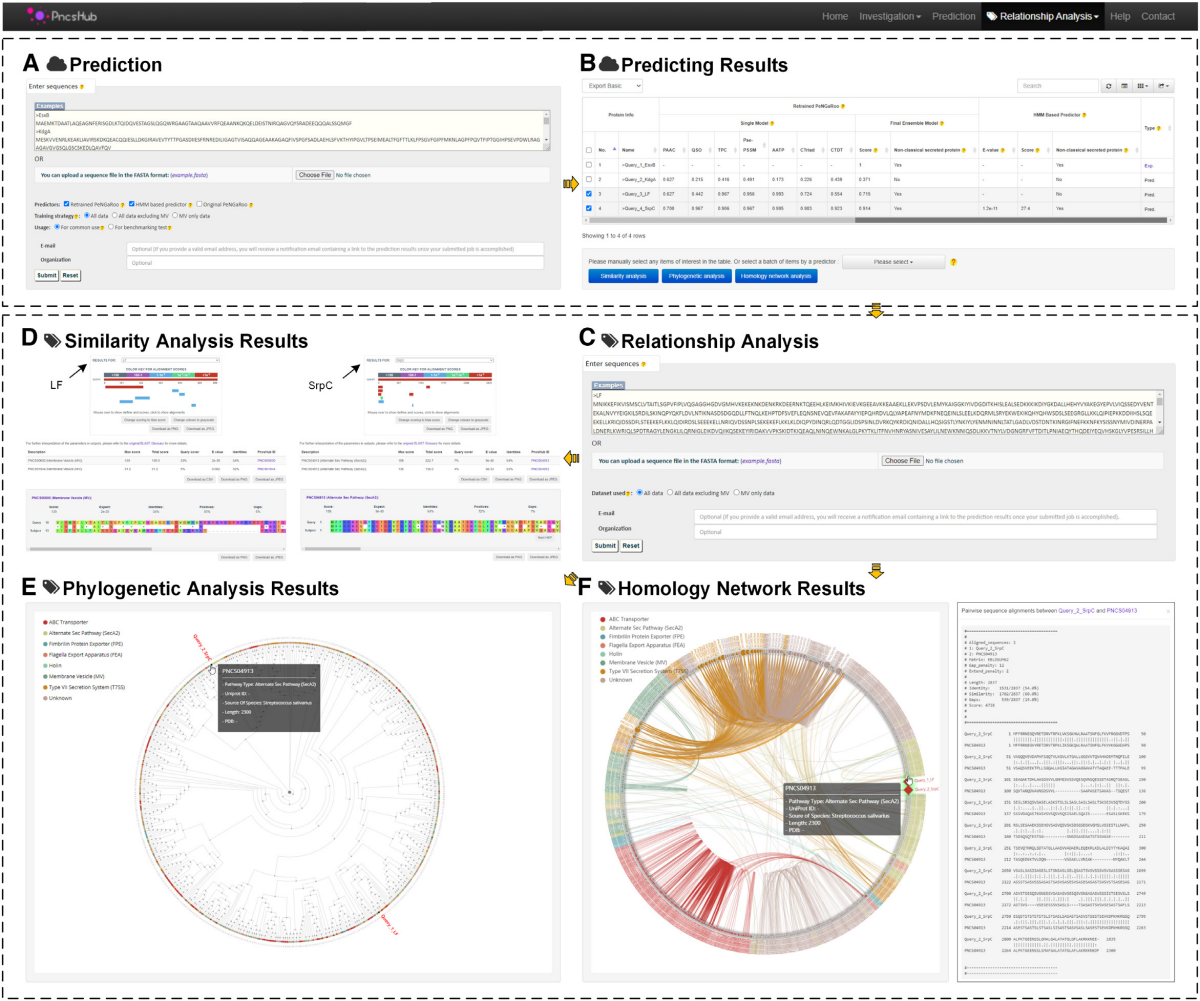
Graphical illustration of the functional modules in PncsHub. (**A**, **B**) Example sequences were submitted as query proteins to both the HMM and retrained PeNGaRoo prediction tools: EsxB (PNCS00367), LF (PNCS00598), SrpC (PNCS04914), and the negative control KdgA (UniProt ID: P50846). EsxB, LF, and SrpC are experimentally validated non-classically secreted substrates (not included into any of our training datasets), but only EsxB is included in the ‘filter list’. (**C**) LF and SrpC were subsequently transferred to our Relationship Analysis Module to determine likely secretion pathways using (**D**) Similarity Analysis, (**E**) Phylogenetic Analysis, and (**F**) Homology Network Analysis.

By selecting these four ‘Example’ sequences in our Prediction Module and submitting them to both the HMM based predictor and the retrained PeNGaRoo predictor (Figure 4A), EsxB was filtered out and selected as an experimentally verified protein, whereas KdgA was correctly predicted as not being a secreted protein by both prediction models (Figure 4B). As test proteins, we noted that only SrpC was correctly predicted to be a non-classically secreted substrate by both models (Figure 4B), whereas LF was only correctly predicted using the retrained PeNGaRoo predictor (Figure 4B). Although neither predictor divulges which secretion pathway LF or SrpC likely uses, our Relationship Analysis Module can allow users to determine the most likely pathway.

We therefore transferred both LF and SrpC to our Relationship Analysis Module (Figure 4C), and observed that, as expected, LF showed limited identity to proteins secreted through MVs (Figure 4D), in particular the related edema factor (EF) component of the anthrax toxin. Furthermore, we observed that SrpC is most likely secreted through the SecA2 apparatus, based on its strong identity to other serine rich proteins that are also secreted in a SecA2-dependent manner (Figure 4D–F). Considering these proof-of-principle results for LF and SrpC, we next sought to apply our Relationship Analysis Module to our list of non-classically secreted proteins that are annotated with an ‘unknown’ secretion mechanism. Overall, we have identified 269 ‘unknown’ proteins in our database, some of which we were able to manually annotate with ‘Possible’ subtypes based on genome context and previous publications (see ‘Data curation and analysis’ section above). Overall, we were able to infer at least one possible subtype for 225 unknown proteins (16 could be annotated with three possible subtypes, 74 could be annotated with two possible subtypes, and 135 could be annotated with 1 possible subtype) (Figure 2, Supplementary Data S1). For the remaining 44 proteins that could not be further annotated, we had previously annotated one of these in the ‘Data curation and analysis’ section (XepA; PNCS00376) as being secreted through a ‘Possible Holin’ (31), and a further five were previously annotated as secreted through a ‘Possible T7SS’ (Supplementary Data S1). Overall, 38 proteins have a bona-fide unknown secretion mechanism suggesting that there are other, as yet undiscovered methods by which bacteria can secrete these proteins.

## DISCUSSION

The spread of virulence factors by horizontal gene transfer separates the most pathogenic bacteria from the rest. And it is the presence of robust and flexible secretion machinery that becomes essential for recipient cells to utilize these ‘alien’ protein sequences (81,82). Recently, there have been several interesting reports of bacteria secreting recombinant proteins (derived from another bacterium) through their own non-classical secretion systems. From *Ruminococcus* sp. 5_1_39BFAA (Gram-positive) but secreted by *Bacillus subtilis* (Gram-positive) (83,84), from *Geobacillus thermoglucosidasius* (Gram-positive) but secreted by *E. coli* (Gram-negative) (85), and from *Ochrobactrum* sp. M231 (Gram-negative) but secreted by *B. subtilis* (86), each new host is capable of secreting these ‘alien’ proteins using their non-classical secretion systems.

PncsHub was developed in order to integrate non-classically secreted substrates from Gram-positive bacteria into a universal database to spur new hypotheses and experiments. PncsHub will be maintained for at least 5 years and will be periodically updated to keep apace with emerging substrates and new experimental details as they become available. Together with the BastionHub database (25), we explore the vast majority of non-classical secretion systems in both Gram-positive and Gram-negative bacteria. Currently, there are seven recognised non-classical protein secretion systems in Gram-positive bacteria, and at least 10 in Gram-negative bacteria (87), but there must be many more considering the range of proteins identified in the secretomes of bacteria (16), and the 38 proteins that remain annotated as ‘Unknown’ after performing comprehensive pathway annotations (Figure 2, Supplementary Data S1). Outside the confines of traditional Gram-positive and Gram-negative bacteria are the Tenericutes that, while bounded by a single membrane, Gram stain negative because they do not contain peptidoglycan. Intriguingly, two reports investigating the secretomes of three *Mycoplasma* species suggest that the majority of proteins secreted by these Tenericutes are through non-classical secretion systems (88,89). In addition to the classical secretion systems that are essential for viability, the non-classical secretion systems play pivotal roles in pathogenesis, cell-to-cell communication, DNA uptake, and motility. Both the knowns and unknowns of bacterial secretion systems are an exciting and rapidly expanding area of research, and it is our hope that Gram-positive and Gram-negative classically and non-classically secreted proteins will ultimately be integrated into an all-in-one universal platform.

## DATA AVAILABILITY

The PncsHub platform is freely available at https://pncshub.erc.monash.edu/. All data within PncsHub can be downloaded via https://pncshub.erc.monash.edu/download.jsp. Detailed user instructions for PncsHub can be accessed via its Help page at https://pncshub.erc.monash.edu/help.jsp.

## Supplementary Material

gkab814_Supplemental_File

## Notes

Present address: Jiawei Wang, European Molecular Biology Laboratory, European Bioinformatics Institute (EMBL-EBI), Wellcome Genome Campus, Hinxton, Cambridge CB10 1SD, UK.
